# Postoperative outcomes after total hip arthroplasty in patients with Parkinson disease: A protocol of case control study

**DOI:** 10.1097/MD.0000000000020018

**Published:** 2020-05-08

**Authors:** Yuerong Zhang, Ke Xiong, Ruizhen Li, Li Yang

**Affiliations:** a Department of Orthopedics; b Department of Clinical Laboratory; c Department of Ophthalmology, People's Hospital of Dujiangyan, Sichuan Province, China.

**Keywords:** complication, Parkinson disease, protocol, revision, total hip arthroplasty

## Abstract

**Background:**

Parkinson disease (PD) is a progressive neuromuscular disease associated with bradykinesia, tremor, and postural instability. We aimed to compare outcomes and complications of total hip arthroplasty (THA) between patients with PD and those without.

**Methods:**

A single institution retrospective cohort from 2000 to 2018 was reviewed. PD patients were matched 1:2 with non-PD control patients for age, gender, American Society of Anesthesiologists score, and body mass index using a propensity score matching procedure. The primary outcome measures were postoperative complications and revision between PD and cohort groups. Secondary outcome measures were Harris Hip Score, hip range of motion, patient satisfaction, and surgery time. Univariable and multivariable logistic regression were used to determine the relationship between PD and surgical outcomes in the matched cohort.

**Results:**

Using prospectively collated data, we identified 35 PD patients after primary THA. A control cohort of 70 primary THA patients was matched.

**Conclusion:**

Our hypothesis was that PD would have adverse impact on complication rates, range of movement, or improvement in functional outcome after subsequent THA.

**Trial registration:**

This study protocol was registered in Research Registry (researchregistry5446).

## 1 Introduction

Parkinson disease (PD) is a progressive neurodegenerative disorder characterized by worsening motor symptoms, including a resting tremor, bradykinesia, rigidity, shuffling gait, and poor overall coordination, all of which significantly increase the risk of falls.^[1,2]^ PD is the second most common neuromuscular disease, affecting 150 to 200 persons per 100,000 and 1% of the population over age 60 years.^[3]^ PD is associated with a variety of orthopedic conditions, including osteopenia and increased risk of fall and fracture. The muscular rigidity and diminished bone quality encountered in PD patients presents important challenges to the orthopedic surgeon.^[4]^

Total hip arthroplasty (THA) is a well-established, safe surgical procedure for end-stage degenerative osteoarthritis that has been found to be effective in reducing pain, increasing range of movement, as well as improving self-reported patient mobility and function for those who do not respond to nonsurgical interventions.^[5,6]^ Previously, PD has been considered a contraindication to both THA and total knee arthroplasty.^[3,7]^ Patients with PD have reportedly experienced higher rates of medical complications than those among the general population.^[8]^ Early studies also demonstrated high postoperative mortality following arthroplasty in PD patients, suggesting increased dislocation rates with uncertain long-term functional gains.^[3]^ However, recent studies have suggested that both hip and knee arthroplasty provide excellent long-term pain relief, with dislocation rates likely comparable to that of the general population.^[9–11]^ In addition, it has been suggested that aggressive rehabilitation and early weight-bearing after surgery decrease the dislocation rate and provide PD patients with functional improvement until around 1 year postoperatively, at which point functional decline appears to result from progression of the neuromuscular disease itself.^[12]^

Therefore, despite the increase in interest in the application of arthroplasty procedures in patients with PD, data on outcomes of these procedures are still relatively scarce. Given the sparse evidence on this topic, the aim of this study is to elucidate the outcomes of THA in patients with PD with respect to survivorship, complications, and functional outcomes in comparison to a cohort group without PD.

## 2 Materials and methods

### 2.1 Patient population

A retrospective review of patients undergoing primary THA at a single institution from 2000 to 2018 was performed. This retrospective cohort study was approved by the institutional review board in our hospital (JY2020042) and was registered in the research registry (researchregistry5446). We identified all patients who underwent primary THA with PD and who had at least 1 year of clinical follow-up for the THA. Patients were excluded for

(1) a history of hip surgery before the primary THA,
(2) previous ipsilateral hip fracture or traumatic injury, and
(3) hip disease secondary to infection or rheumatologic disorders.

Patients in the study group were matched 1:2 with patients in the cohort group based on the following criteria: age at time of THA (±3 years), body mass index (±3 points), sex, and American Society of Anesthesiologists score (±1 point).

### 2.2 Techniques

Indications for THA were severe hip pain and/or considerable difficulty in walking and performing daily activities with significant hip osteoarthritic changes, complete hip joint dislocation, or an ankylosed hip joint. All THAs in this study were carried out by either the senior author, or by fellows under his direct supervision. A tourniquet was used in all cases, general anesthesia was administered to each patient before incision, and the operative hip was prepared and draped in a conventional sterile fashion.

### 2.3 Postoperative care

Postoperative drainage lasts 1 to 2 days until flow volume is less than 30 ml. All patients received the same standardized postoperative multimodal pain protocol, with four doses of 1 g of acetaminophen, 2 doses of celecoxib 200 mg, and morphine (first 48 hours) or tramadol (after 48 hours) for pain exacerbations. All patients underwent the same postoperative rehabilitation program, with partial weight bearing with the use of crutches for the first postoperative day and active range of movement exercises.

### 2.4 Outcome variables

The patient demographics, American Society of Anesthesiologists Score (ASA) grade, and body mass index were recorded retrospectively from their electronic patient notes. The primary outcome measures were postoperative complications and revision between PD and cohort groups. Secondary outcome measures were Harris Hip Score, hip range of motion, patient satisfaction, and surgery time. All complications, surgery time, as well as need for revision surgery, were obtained from review of the electronic medical records. All data were independently verified by a detailed review of hospital operative reports, anesthesia records, and clinical records. Range of motion, Harris Hip Score and patient satisfaction were obtained both before and after arthroplasty by a dedicated physiotherapist specialist. Harris Hip Score was originally developed to evaluate the treatment of post-traumatic arthritis, but is now widely used for any osteoarthritis of the hip, and has been found to be responsive for these patients. The score has a maximum of 100 points (best possible function), covering pain (1 item, 0–44 points), function and activities (7 items, 0–47 points), and range of motion and absence of deformity (3 items, 0–9 points). Patient satisfaction was assessed by asking the question “How satisfied are you with your operated hip?” one year after surgery. The response was recorded using a five point Likert scale: very satisfied, satisfied, neutral, unsatisfied and very unsatisfied. Patients who recorded very satisfied or satisfied were classified as satisfied. For patients who were not seen recently, the scores were obtained via telephone.

### 2.5 Statistical analysis

Parkinson and control groups were compared using chi-squared test to compare qualitative variables between groups. The Student unpaired *t* test was used to compare qualitative outcomes between groups. All statistical analyses were performed using the SPSS software program for Windows (version 22; IBM Corp., Armonk, NY). A *P* value of less than .05 was considered to be statistically significant. Univariable and multivariable logistic regression were used to determine the relationship between PD and surgical outcomes in the matched cohort.

## 3 Result

Using prospectively collated data, we identified 35 PD patients after primary THA. A control cohort of 70 primary THA patients was matched. The results will be shown in Tables 1–3.

**Table 1:**
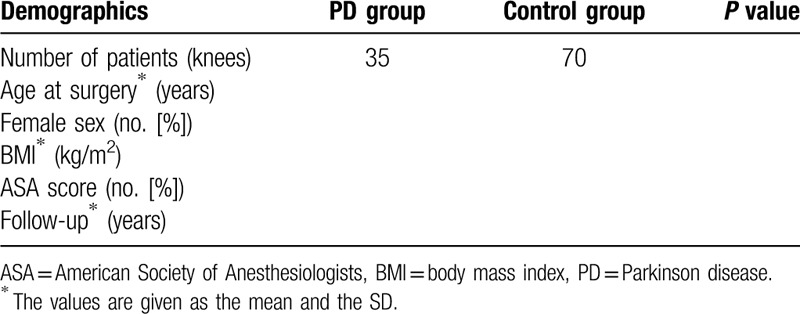
Patient baseline demographics.

**Table 2:**
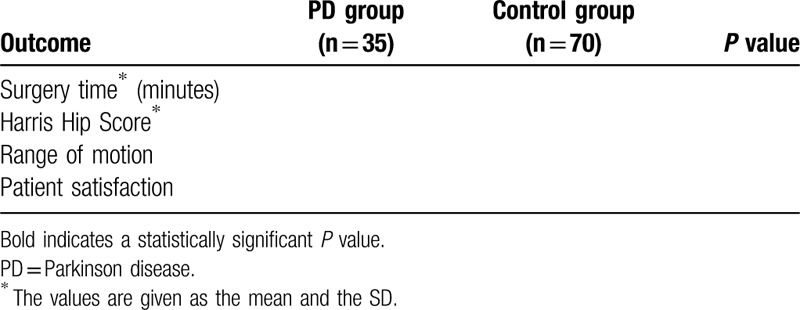
Intraoperative and functional outcomes.

**Table 3:**
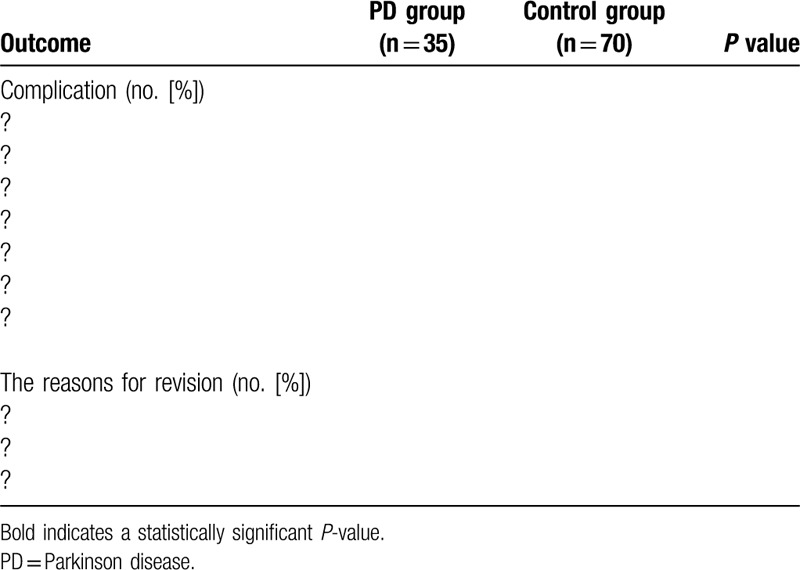
The compications and revision in the two groups.

## 4 Discussion

PD is one of the most common neurological disorders in patients who undergo THA. The relationship between PD and orthopedic injury has been studied in the literature. A systematic review performed by Allen et al reported that 60.5% of patients with PD will experience falls as a result of disease symptoms, and subsequently 39% will experience recurrent falls.^[13]^ One study retrospectively compared the risk of falls/facture between non-PD patients and PD patients, and found an overall adjusted incidence rate ratio of 2.05 (95% CI 1.88–2.24), indicating that the incidence of falls/fractures was significantly higher in subjects with PD compared to non-PD subjects in the US population.^[14]^ Several studies have also identified hip fractures as the most common fracture experienced by this patient population.^[15,16]^ In PD patients who undergo spine surgery, Schroeder et al reported that patients with PD demonstrated an increased risk of revision, but significant pain relief and improved SF-12 scores.^[17]^

Knowledge of the impact of PD on the outcomes of a subsequent THA, however, is currently limited. A concern exists that PD may lead to more challenging surgery and the subsequent sequelae of complications that this entails, as has previously been highlighted. The primary purpose of this study was to investigate the impact of PD on postoperative THA complications and revision. Our hypothesis was that PD would have adverse impact on complication rates, range of movement, or improvement in functional outcome after subsequent THA.

The limitations of our study included those inherent in any retrospective cohort study, including the possibility of selection or observational bias. This study also did not address long-term follow-up (10 years) as our study relied on electronic medical records kept since 2011. The authors recognize that longer term follow-up is critical in determining the influence of PD on THA specifically on infection, implant loosening, revision, and long-term function outcomes. Additionally, although we performed a matched study based on age, gender, American Society of Anesthesiologists score, and body mass index, it is likely that there were other preoperative features that we could have controlled for that may have led to alternative results.

## Author contributions

Yuerong Zhang and Ke Xiong conceived, designed, and planed the study. Yuerong Zhang, Ruizhen Li, and Ke Xiong are recruiting the study participants and performing the interventions. Ke Xiong supervised the study. Yuerong Zhang, Li Yang, and Ruizhen Li will interpret and analyze the data. Yuerong Zhang drafted the manuscript. Ke Xiong critically revised the manuscript for important intellectual content. All authors have full access to the manuscript and take responsibility for the study design. All authors have approved the manuscript and agree with submission.
